# Expression profile, molecular functions, and prognostic significance of miRNAs in primary colorectal cancer stem cells

**DOI:** 10.18632/aging.202914

**Published:** 2021-04-01

**Authors:** Chuan-Wen Fan, Ran Lu, Chao Fang, Xue-Li Zhang, Zhao-Ying Lv, Yuan Li, Hong Zhang, Zong-Guang Zhou, Xian-Ming Mo, Xiao-Feng Sun

**Affiliations:** 1 Institute of Digestive Surgery, Sichuan University, and Department of Gastrointestinal Surgery, West China Hospital, West China School of Medicine, Sichuan University, Chengdu, China; 2 Department of Gastrointestinal Surgery and Breast and Thyroid Surgery, Minimally Invasive Surgery, West China Fourth Hospital, Sichuan University, Chengdu, China; 3 Department of Oncology and Department of Biomedical and Clinical Sciences, Linköping University, Linköping, Sweden; 4 Laboratory of Stem Cell Biology, West China Hospital, Sichuan University, Chengdu, China; 5 School of Medicine, Institute of Medical Sciences, Örebro University, Örebro, Sweden

**Keywords:** miRNAs, cancer stem cells, progression, prognosis, colorectal cancer

## Abstract

MicroRNAs (miRNAs) are known to drive the pathogenesis of colorectal cancer (CRC) via the regulation of cancer stem cells (CSCs). We studied the miRNA expression profile of primary CSCs isolated from patients with CRC (pCRCSCs). Compared to pCRCSC-derived differentiated cells, 98 differentially expressed miRNAs were identified in pCRCSCs. Target genes encoding pCRCSC-related miRNAs were identified using a combination of miRNA target databases and miRNA-mRNA regulatory networks from the same patient. The pCRCSC-related miRNA target genes were associated with pathways contributing to malignant phenotypes, including I-kappa B kinase/NF-kappa B signaling, signal transduction by p53 class mediator, Ras signaling, and cGMP-PKG signaling. The pCRCSC-related miRNA expression signature was independently associated with poor overall survival in both the training and validation cohorts. We have thus identified several pCRCSC-related miRNAs with oncogenic potential that could serve as prognostic biomarkers for CRC.

## INTRODUCTION

Colorectal cancer (CRC) is one of the leading causes of cancer-related death worldwide due in large part to its strong recurrence and metastatic potentials [1]. Cancer stem cells (CSCs), a subset of cancer cells, are characterized by sphere formation, self-renewal, and multi-lineage differentiation, and contribute to cancer initiation and metastasis and resistance to chemotherapy, radiotherapy, and targeted therapy in various cancer types, including CRC [2]. We previously isolated and identified primary rectal CSCs and showed their characteristic phenotypes [3]. Further studies indicated that these primary CSCs can transdifferentiate into endothelial cells and neurons to support the growth of CRC cells [4, 5]. Compared with CSCs derived from cancer cell lines, primary CSCs are practically more relevant and reflect the actual tumor conditions in cancer patients [6]. However, a detailed understanding of malignant characteristics of primary CSCs isolated from patients with CRC (pCRCSCs) and the underlying molecular mechanisms of development of CSCs is required to mimic the CSC characteristics *in vivo*.

MiRNAs are small non-coding RNAs of approximately 20 to 25 nucleotides in length that regulate the expression of more than 60% of human genes. Recent findings have implicated several miRNAs, such as miR-21 [7], miR-27a [8], miR-31 [9], miR-137 [10], miR-146a [11], miR-148a [12], miR-195-5p [13], miR-196b-5p [14], miR-199a/b [15], miR-215 [16], miR-372/373 [17], and miR-1246 [18], in the regulation of CSC characteristics including self-renewal and differentiation [19, 20]. MiRNAs execute these functions by targeting the genes of several essential signaling pathways, such as Wnt/β-catenin and Notch, associated with the maintenance, growth, and function of CSCs [21]. However, a global miRNA expression profile of CRCSCs, especially of pCRCSCs is still unavailable. Moreover, CSC-related signaling pathways do not function in isolation but as a coordinated network [22, 23], implying that CSC phenotypes are an output of several signaling networks. Similarly, a single miRNA can target multiple genes and signaling pathways. Therefore, a single agent targeting the rare CSC subpopulations, although can reduce the tumor volume, cannot eliminate the tumor, highlighting the need to elucidate the regulatory network of pCRCSCs.

**Figure 1:**
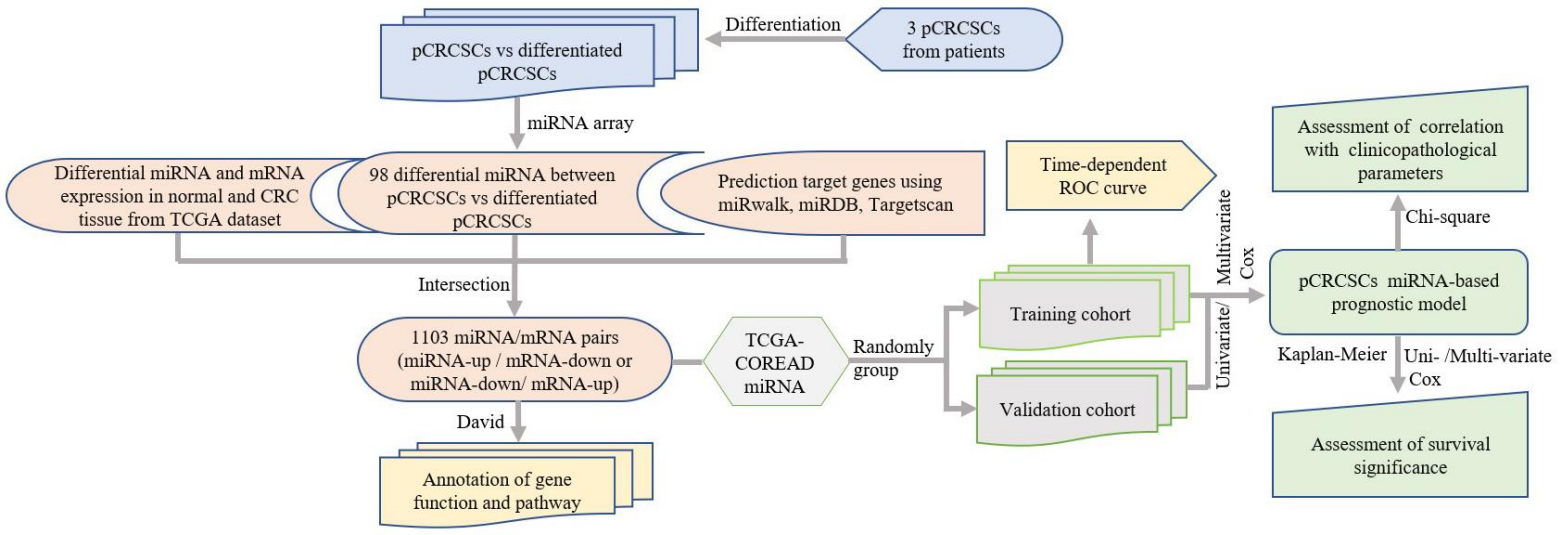
**Study design.** pCRCSCs: primary colorectal cancer stem cells.

We performed a comprehensive global miRNA expression analysis of differentiated pCRCSCs to identify differentially expressed miRNAs. Further, molecular functions of differentially expressed miRNAs were annotated and their prognostic significance in patients with CRC was analyzed. The study design is shown in Figure 1.

## RESULTS

### Expression profiles of miRNAs in pCRCSCs and their corresponding pCRCSC-derived differentiated cells

**Figure 2:**
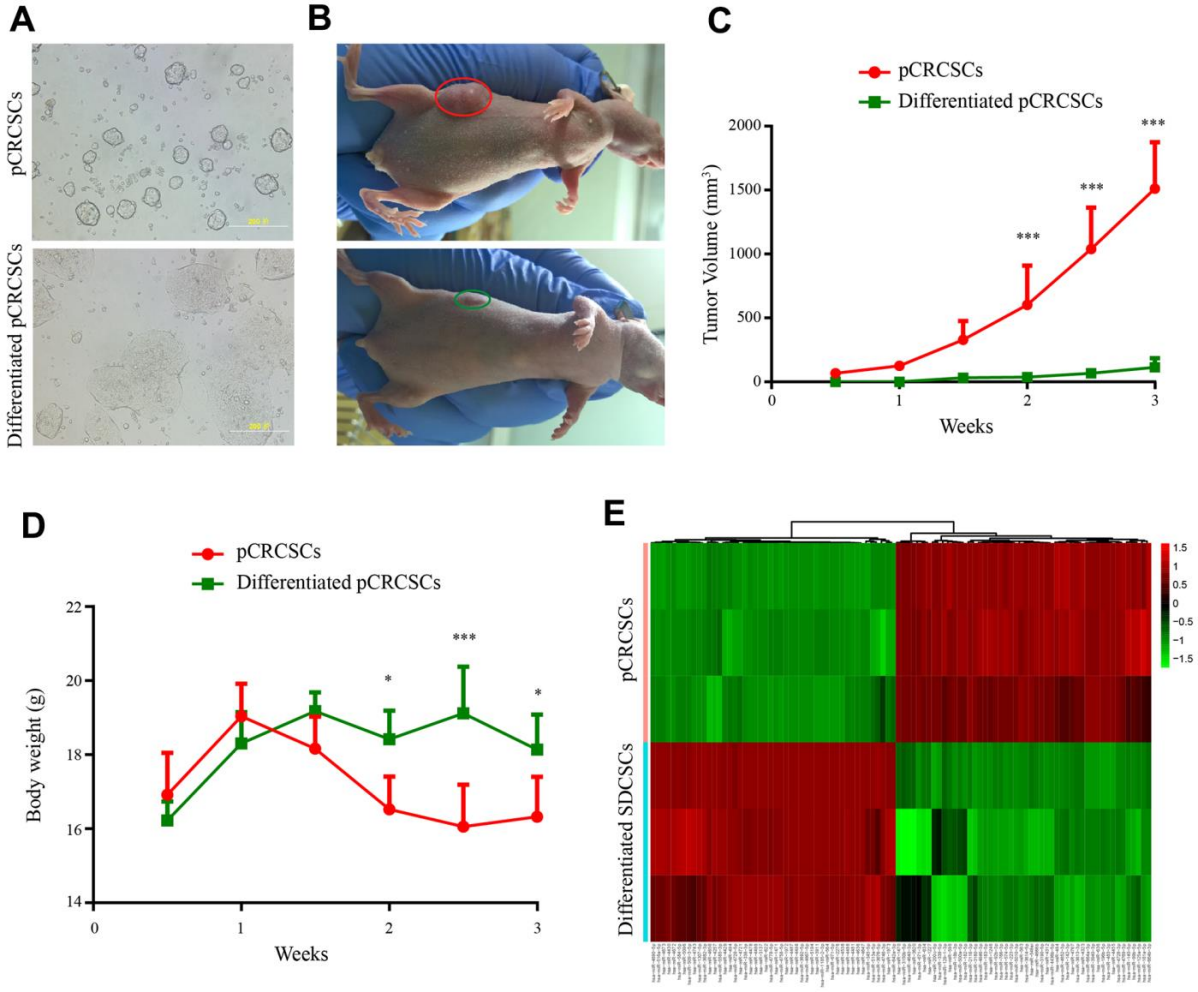
**Differential tumorigenic capacity and distinct expression profiles of miRNAs between pCRCSCs and pCRCSCs-derived differentiated cells.** (**A**) pCRCSCs generated from a human colon cancer sample and their corresponding differentiated pCRCSCs. Bars = 200 μM. (**B**) A tumor-bearing nude mouse showing a large xenograft tumor from pCRCSCs (red oval) and a small tumor from pCRCSC-derived differentiated cells (green oval). (**C**) Tumor volume of the subcutaneous tumor generated from 5 × 10^5^ pCRCSCs and differentiated pCRCSCs. (**D**) Weight of tumor-bearing mice. Bars represent mean ± standard deviation (SD) (*n* = 5, ^*^p < 0.05, ^***^p < 0.001). (**E**) Heat map of differentially expressed miRNAs in pCRCSCs and pCRCSC-derived differentiated cells.

To isolate colon CSCs, primary CSCs from patients with CRC were enriched in serum-free medium supplemented with epidermal growth factor (EGF) and basic fibroblast growth factor (bFGF). After 3 to 4 weeks, a small fraction of tumor cells formed spheres (Figure 2A). Next, the serum-free CSC medium was replaced with 20% fetal bovine serum (FBS)-containing medium to differentiate the spheres [3, 24]. The spherical cells in the culture gradually aggregated into clusters of polygonal cells and exhibited typical epithelial-like tumor cell morphology (Figure 2A).

To study tumorigenesis between the expanded pCRCSCs and pCRCSC-derived differentiated cells (termed differentiated pCRCSCs in the following text), both cell types were injected into immunodeficient mice and xenograft growth was assessed. Compared to differentiated pCRCSCs, pCRCSCs formed bigger tumor masses, with a faster growth (Figure 2B, 2C). In addition, mice-bearing pCRCSCs lost more weight than those bearing differentiated pCRCSCs (Figure 2D).

To further investigate the difference in the potential miRNAs regulating tumorigenesis between pCRCSCs and differentiated pCRCSCs, a global miRNA expression profile analysis was performed in three pCRCSCs and paired differentiated pCRCSCs including one primary CSC isolated from a patient with CRC and two previously enriched primary CSCs isolated from patients with rectal cancer [3]. In total, 98 differentially expressed miRNAs were identified between pCRCSCs and differentiated pCRCSCs, of which 50 were upregulated and 48 were downregulated (Figure 2E and Supplementary Table 1).

### Identification of target genes and regulatory network of pCRCSC-related miRNAs

**Figure 3:**
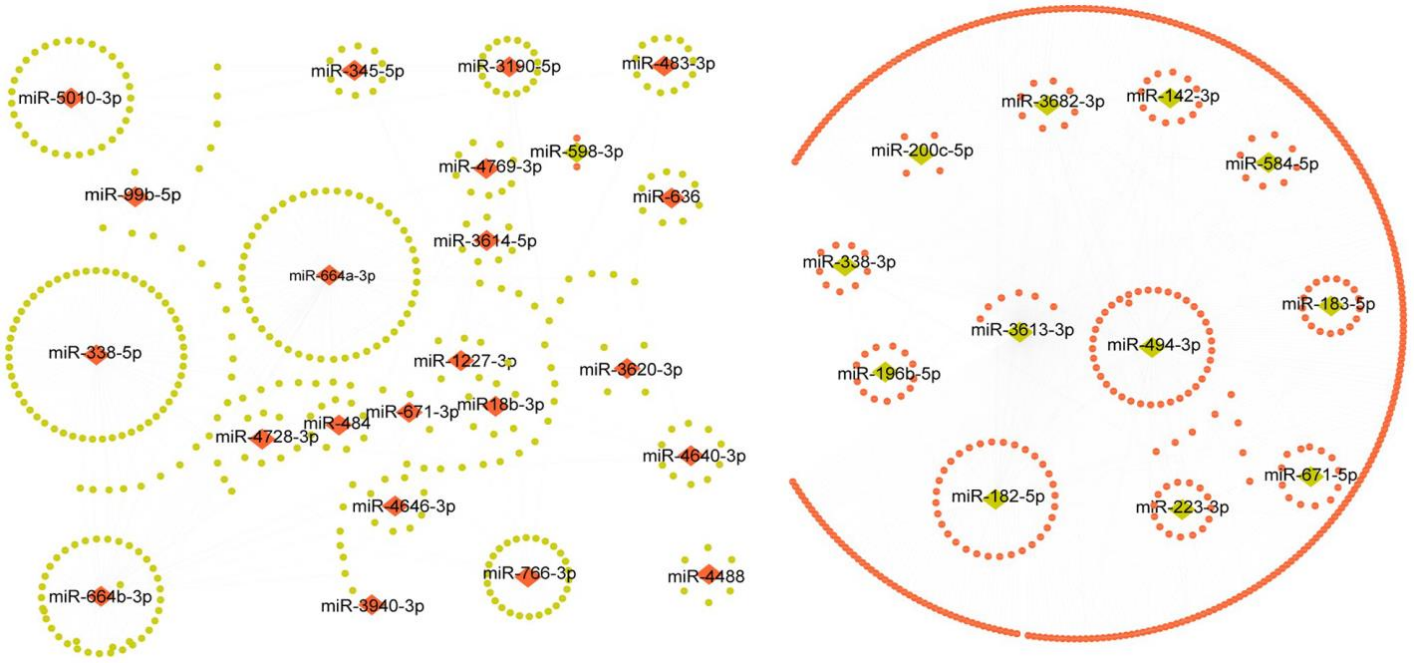
**The regulatory network of pCRCSC-related miRNAs.** Upregulated miRNAs are shown as red diamonds and the corresponding downregulated target genes in the TCGA database are shown as green ellipses. The downregulated miRNAs are shown as green diamonds and the corresponding upregulated target genes in the TCGA database are shown as red ellipses.

Because miRNAs function by binding to their specific target genes, we first used miRWalk, TargetScan, and miRDB databases to predict target genes for all 98 differentially expressed miRNAs in pCRCSCs. Only genes that were commonly predicted by all three databases were used as putative target genes. In total, 18,792 potential target genes were identified (Supplementary Table 2). To more accurately identify the miRNA targets, we analyzed the miRNA and mRNA differential expression profile datasets of the same patients using the TCGA database. We identified 745 miRNAs and 5,558 mRNAs (|log2 FC| > 2, p < 0.05) that were differentially expressed in normal and CRC samples (Supplementary Tables 3, 4). The negatively regulated miRNA/mRNA pairs (miRNA upregulated/mRNA downregulated or miRNA downregulated/mRNA upregulated) were obtained and intersected with predictive target mRNAs in the database. The 1,103 miRNA/mRNA pairs were identified, including 35 miRNAs and 870 mRNAs, were believed to be related to miRNA-regulated target genes in pCRCSCs (Supplementary Table 5). The miRNA–mRNA regulatory network is shown in Figure 3.

### Relevant biological functions and pathways affected by pCRCSC-related miRNAs

**Figure 4:**
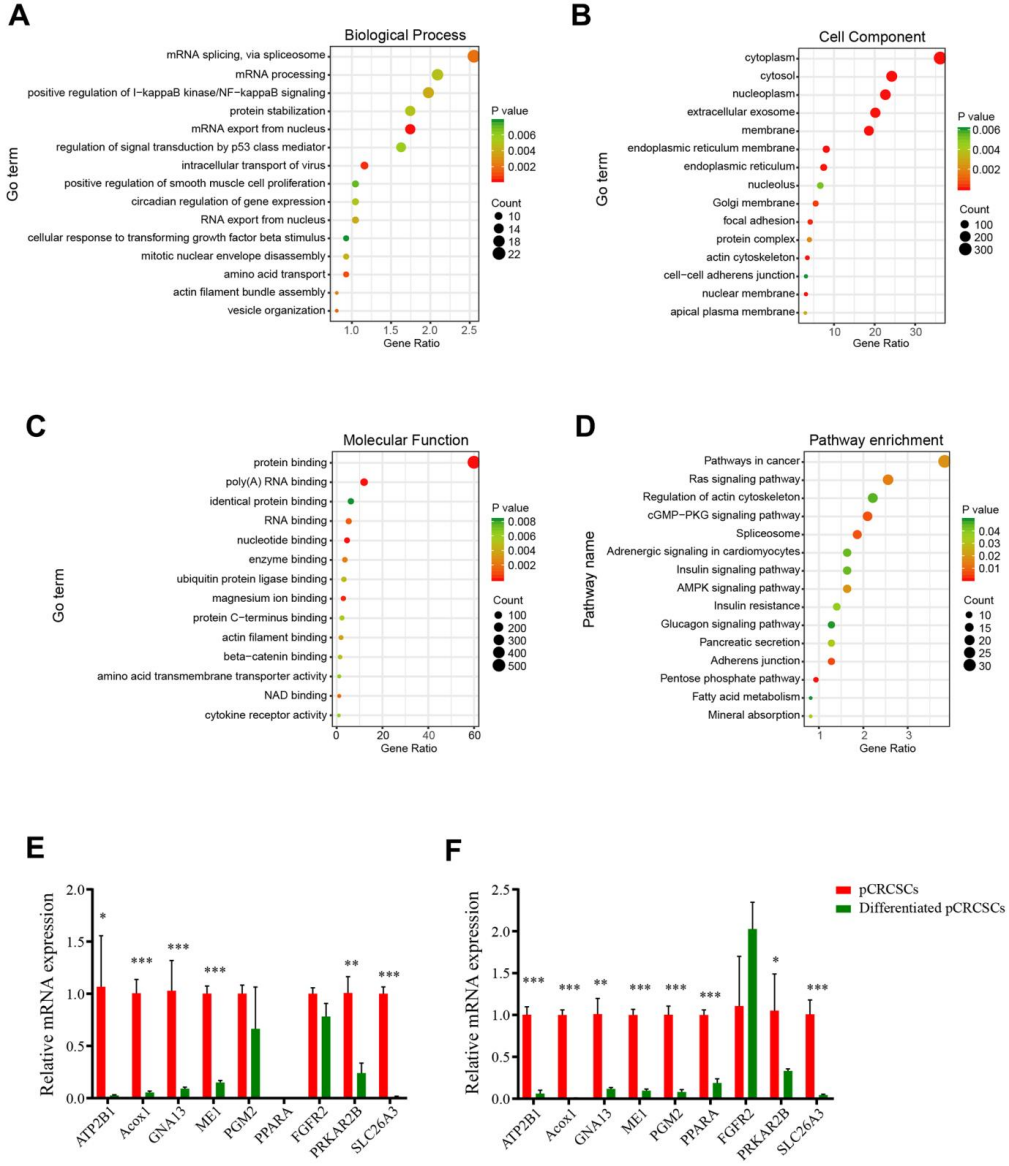
**The GO and KEGG pathways of pCRCSC-related miRNAs.** (**A**) The top 15 biological processes, (**B**) cell components, (**C**) molecular functions, and (**D**) the KEGG pathways of target genes based on the intersection of predicted downstream target genes and genes that negatively correlated with miRNA expression in the TCGA–COREAD dataset. (**E**) Nine potential target genes of pCRCSC-related miRNAs were validated by RT-qPCR in the primary CSCs derived from colon cancer and the corresponding differentiated cells. (**F**) Nine potential target genes of pCRCSC-related miRNAs were validated by RT-qPCR in the primary CSCs derived from rectal cancer and the corresponding differentiated cells. GAPDH was selected as the internal control. This experiment was repeated thrice. Bars represent mean ± standard deviation (SD) (*n* = 3, ^*^p < 0.05, ^**^p < 0.01, ^***^p < 0.001).

To assess the biological functions of pCRCSC-related miRNAs, the functions and pathways of targeted genes of pCRCSC-related miRNAs were analyzed using the DAVID database. In total, 868 genes were found enriched in 68 gene ontology (GO) terms including 23 biological processes, 31 cell components, and 14 molecular functions (Figure 4A–4C and Supplementary Table 6). The enriched biological processes included mRNA splicing regulation, I-kappa B kinase/NF-kappa B signaling, and signal transduction by p53 class mediator, which were linked to malignant features of CSCs. Protein binding, poly(A) RNA binding, nucleotide binding, and ubiquitin protein ligase binding were highly enriched molecular functions.

The pCRCSC-related miRNAs were enriched in several cancer-related pathways, including Ras signaling pathway, actin cytoskeleton regulatory pathways, cGMP–PKG signaling pathway, and spliceosome pathways, which correlated with the malignant phenotype of cancer cells (Figure 4D and Supplementary Table 7).

Because pCRCSC-related miRNA targets were predicted by bioinformatics, certain potential targets involved in the key pathways were selected and examined by quantitative reverse transcription-polymerase chain reaction (RT-qPCR) for validation. The expression of selected potential targets was congruent with the results of predictive miRNA target databases, validating the involvement of predictive miRNA-related signaling pathways in CRC (Figure 4E, 4F).

### Identification of potential prognostic miRNA signatures for CRC

To evaluate the prognostic function of pCRCSC-related miRNAs in patients with CRC, we first randomly grouped the TCGA–COREAD data into training and validation cohorts (Supplementary Table 8). The prognostic significance of 35 functionally annotated pCRCSC-related miRNAs was further investigated using univariate Cox proportional hazards regression analyses in the training cohort (Supplementary Table 9). Prognosis-related miRNAs were subsequently selected for multivariate Cox proportional hazards regression analyses. Finally, two pCRCSC-related miRNAs (miR-664b-3p [risky miRNA] and miR-200c-5p [protective]) were confirmed as independent prognostic miRNAs of patients with CRC in the training cohort (Supplementary Table 10). To facilitate the use of pCRCSC-related miRNAs as prognostic markers in routine clinical practice, we next developed a formula to calculate the risk score of overall survival (OS) using the Cox proportional hazard regression model for each patient based on the expression of two pCRCSC-related miRNAs, where risk score = (0.384 × expression of miR-664b-3p) – (0.270 × expression of miR-200c-5p).

### Clinical significance of prognostic miRNA signature in CRC patients

**Figure 5:**
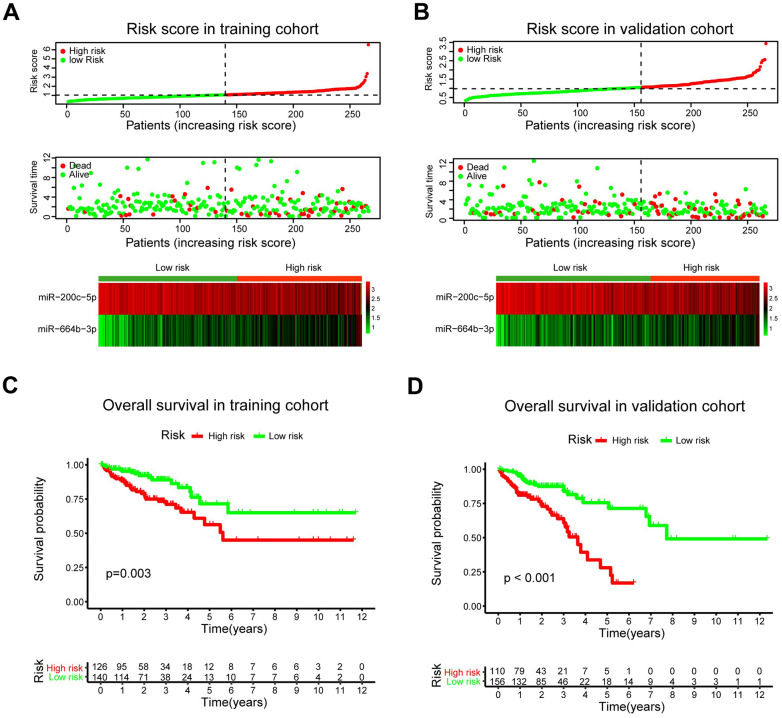
**A prognostic model based on pCRCSC-related miRNA signature stratifies the OS in CRC patients.** The distribution of risk score, overall survival (OS), OS status, and the heat map of prognostic pCRCSC miRNA signature in the training (**A**, **B**) validation cohorts. The dotted line indicates the cut-off point of the median risk score used to stratify the patients into low- and high-risk groups. Kaplan–Meier curves of OS for patients with CRC based on pCRCSC-related miRNA signature in the training (**C**, **D**) validation cohorts.

The patients were classified into low- and high-risk groups in the training and validation cohorts using X-tile plots to generate the optimal cut-off score (Supplementary Figure 1). Correlation analysis of clinicopathological characteristics of patients between high- and low-risk groups revealed remarkable differences only in the stage and survival status of patients in both training and validation cohorts (Table 1). In addition, as shown in Figure 5A, 5B, the distribution of miRNA-based risk scores, OS time, OS status, and the expression of two pCRCSC-related miRNAs in the training and validation cohorts showed that high-risk patients were associated with higher mortality than low-risk patients. The heat map revealed that the risky miR-664b-3p was highly expressed in high-risk patients, whereas protective miR-200c-5p was highly expressed in low-risk patients in both training and validation cohorts (Figure 5A, 5B).

**Table 1:** Baseline characteristics of patients by miRNA assessment cohorts.

**Parameters**	**Training set**				**Validation set**		
	**Low risk**	**High risk**	**p value**		**Low risk**	**High risk**	**p value**
**Gender**							
Female	67 (43.8)	56 (49.6)	0.351		73 (45.9)	53 (49.5)	0.562
Male	86 (56.2)	57 (50.4)			86 (54.1)	54 (50.5)	
**Age (years)**							
≤65	68 (44.4)	55 (48.7)	0.494		69 (43.4)	49 (45.8)	0.700
>65	85 (55.6)	58 (51.3)			90 (56.6)	58 (54.2)	
**Tumor location**							
RSCC	81 (52.9)	39 (34.5)	0.004		65 (40.9)	43 (40.2)	0.959
LSCRC	67 (43.8)	68 (60.2)			91 (57.2)	61 (57.0)	
NA	5 (3.3)	6 (5.3)			3 (1.9)	3 (2.8)	
**Stage**
Stage I	31 (20.3)	12 (10.6)	**0.032**		35 (22.0)	15 (14.0)	**0.001**
Stage II/III	103 (67.3)	79 (69.9)			106 (66.7)	63 (58.9)	
Stage IV	15 (9.8)	20 (17.7)			15 (9.4)	28 (26.2)	
NA	4 (2.6)	2 (1.8)			3 (1.9)	1 (0.9)	
**MSI status**							
MSS	118 (77.1)	107 (94.7)	<0.001		133 (83.6)	100 (93.5)	0.069
MSI	35 (22.9)	6 (5.3)			21 (13.2)	7 (6.5)	
NA					5 (3.1)		
**Radiochemotherapy**							
No	90 (58.8)	58 (51.3)	0.224		109 (68.6)	58 (54.2)	0.018
Yes	63 (41.2)	55 (48.7)			50 (31.4)	49 (45.8)	
**Survival status**							
Alive	134 (87.6)	82 (72.6)	**0.002**		134 (84.3)	68 (63.6)	**<0.001**
Dead	19 (12.4)	31 (27.4)			25 (15.7)	39 (36.4)	
**Recurrence**							
No	103 (67.3)	77 (68.1)	0.69		113 (71.1)	67 (62.6)	0.049
Yes	29 (19.0)	19 (16.8)			21 (13.2)	24 (22.4)	
NA	21 (13.7)	17 (15.0)			25 (15.7)	16 (15.0)	

Note: NA: Missing data; RSCC: right-sided colon cancer; LSCRC: left-sided colorectal cancer. Bold values indicate the statistical significance parameters in both training and validation sets (P<0.05).

Survival analyses showed that the 5-year OS was 56.3% (95% confidence interval [CI]: 42.7–74.2) for high-risk patients and 71.5% (95% CI: 57.8–88.3) for low-risk patients (Figure 5C; *p* = 0.003) after assessing the prognostic accuracy of two miRNA-based classifiers with time-dependent ROC analysis at varying follow-up times (Supplementary Figure 2A, 2B). The classifier universality of two pCRCSC-related miRNAs in different populations was confirmed by applying it to the validation cohort, thereby classifying 110 (41%) patients as high risk and 156 (59%) patients as low risk. Five-year OS was 28.1% (95% CI: 14.6–54.1) for high-risk patients and 71.4% (95% CI: 59.3–81.6) for low-risk patients (Figure 5D; *p* < 0.001).

### Assessment of independent prognostic significance of miRNA signature in CRC patients

**Figure 6:**
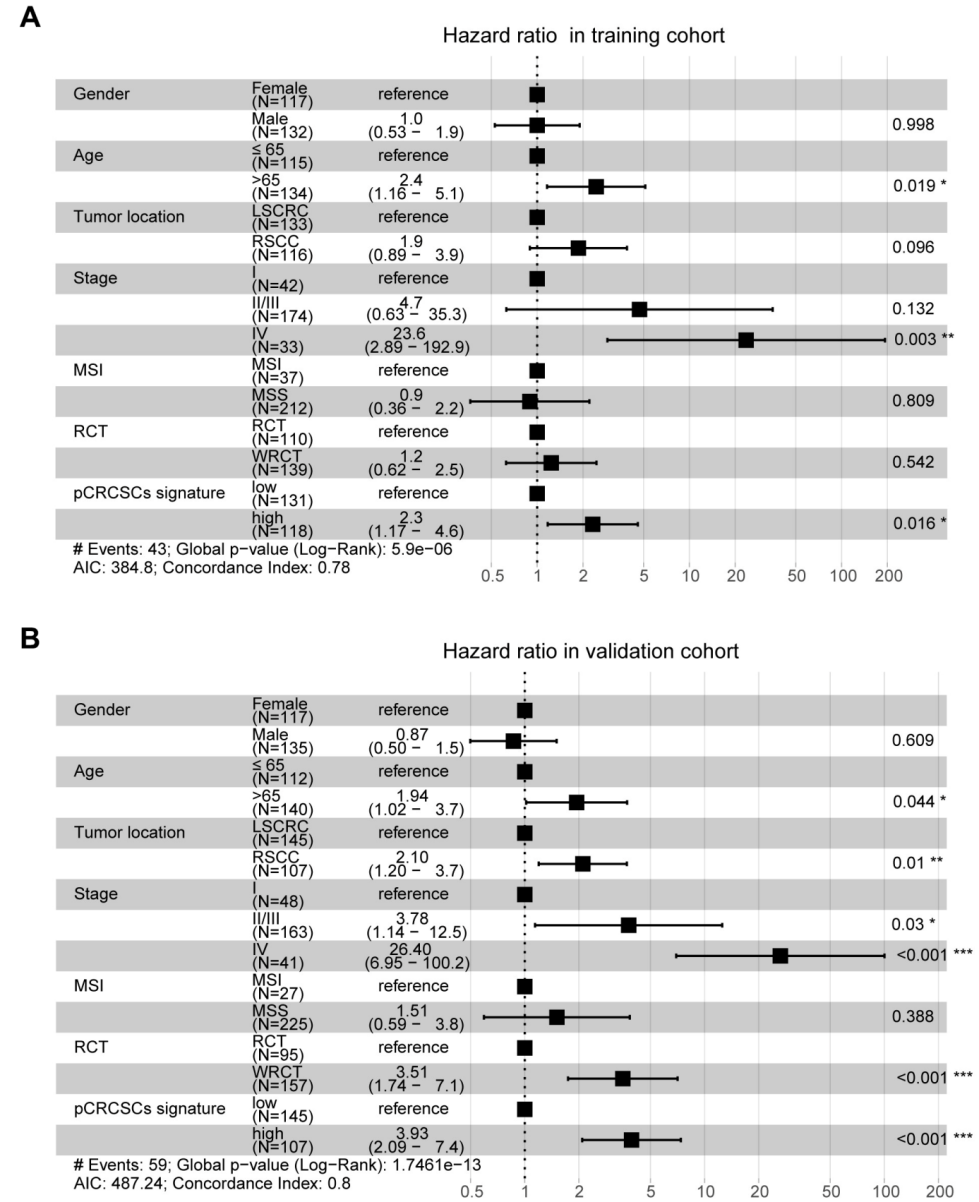
**pCRCSC-related miRNA signature is an independent prognostic factor for OS in CRC patients.** Forest plot summary of multivariate analyses for OS with gender, age, tumor location, tumor stage, MSI status, adjuvant chemoradiotherapy as covariates, and the risk based on pCRCSC-related miRNA signature in the training (**A**, **B**) validation cohorts. Squares on the transverse lines represent the hazard ratio (HR), whereas transverse lines represent 95% confidence interval (CI).

To further assess whether the pCRCSC-related miRNA signature could independently predict OS in patients with CRC, both univariate and multivariate Cox regression analyses were performed by adjusting for gender, age, tumor location, stage, microsatellite instability (MSI) status, and adjuvant chemoradiotherapy as covariates. Univariate analyses revealed that the pCRCSC-related miRNA signature was significantly associated with OS (Supplementary Table 11; hazard ratio [HR] = 2.17, *p* = 0.015). Moreover, the pCRCSC-related miRNA signature was significant, even after adjusting for other covariates in the training cohort (Figure 6A, HR = 2.30, 95% CI: 1.17–4.60, *p* = 0.016). Similarly, the prognostic significance of pCRCSC-related miRNA signature with OS was validated in the validation cohort (Supplementary Table 12; HR = 3.71, *p* < 0.001). Multivariate analyses revealed that the pCRCSC-related miRNA signature remained a powerful prognostic factor in the validation cohort after adjusting for other covariates (Figure 6B, HR = 3.93, 95% CI: 2.09–7.40, *p* < 0.001).

## DISCUSSION

Tumor resistance to traditional chemotherapy and radiotherapy is partially attributed to CSCs and results in cancer recurrence and metastasis [25]. We have previously shown that CSCs from primary rectal adenocarcinoma have a strong tumorigenic capacity and are resistant to common therapeutic drugs used for treating patients with advanced or metastatic CRC [3]. In this study, we further evaluated the tumorigenesis of CSCs of primary colon adenocarcinoma that exhibited strong tumorigenic potential, indicating the common malignant features of primary CSCs of both colon and rectal cancers.

Altered miRNA expression has been shown to contribute to the malignant behavior of CSCs [26]. A comprehensive analysis of the miRNA expression profile revealed 98 differentially expressed miRNAs in pCRCSCs, of which 50 were upregulated and 48 were downregulated. Some of these miRNAs, such as miRNA 200c [27, 28], miR-1246 [29], and miR-494 [30], are involved in the regulation of CSCs. For example, miR-1246 activates the Wnt/β-catenin pathway by inhibiting the expression of Axin-2 and GSK-3β to maintain the stemness of CSCs, including self-renewal, drug resistance, and tumorigenicity [29]. Similarly, miR-494 inhibits BMI-1 expression and prevents self-renewal of breast CSCs/progenitor cells [30]. Furthermore, miRNAs regulate four major signal transduction pathways that affect CSC characteristics: Wnt/β-catenin, BMI-1, Notch, and Hedgehog, and the corresponding pathways [8]. Therefore, we not only confirmed the potential significance of previously published miRNAs in CSCs but also identified certain novel miRNAs affecting the malignant features of pCRCSCs.

Pathway enrichment analysis revealed differentially expressed pCRCSC-related miRNAs to be enriched in I-kappa B kinase/NF-kappa B signaling, signal transduction by p53 class mediator, Ras signaling pathway, actin cytoskeleton regulation, cGMP-PKG signaling pathway, and spliceosome pathways, which are known to correlate with the malignant phenotype of cancer cells [31–33]. Moreover, the majority of pathways reported to be related to pCRCSC-related miRNAs have functional implications in CSCs. For instance, p53 and Ras signaling pathways regulate stem cell differentiation and self-renewal [23, 34]. In addition, p53 regulates certain stem factors or miRNAs, including Bmi-1 and miR-34 [35]. Similarly, the NF-κB pathway has been implicated in inflammation, self-renewal, and maintenance and metastasis of CSCs [36]. Interestingly, a cross-talk among these CSC-related signaling pathways has been reported [22, 23, 37]. Further, univariate and multivariate Cox regression analyses revealed an association between pCRCSC-related miRNA expression and survival. MiR-664b-3p and miR-200c-5p were identified as independent prognostic biomarkers in patients with CRC. MiRNA-200c is a well-studied miRNA in a variety of tumors, including CRC, due to its involvement in epithelial-mesenchymal transition and drug resistance [28]. In addition, miRNA-200c is associated with patient clinicopathology and prognostic significance in certain specific cancer types [38–40]. Integration of two prognosis-related pCRCSC miRNAs into a pCRCSC miRNA signature by risk score method, based on their expression and relative contribution, successfully categorized patients into high- and low-risk groups with large differences in OS. Furthermore, risk stratification showed a pCRCSC miRNA-based classifier as a strong prognostic factor that complements clinicopathological features and MSI status, thereby indicating the function of pCRCSC-related miRNAs in predicting the survival of CRC patients. A limitation of our study was that we only used the TCGA dataset due to the unavailability of the expression data of miR-664b-3p and miR-200c in other public databases. Nevertheless, because TCGA is a reliable database and our study included more than 500 patients, miRNAs identified as specifically expressed in CSCs can be used as promising biomarkers to predict the survival of cancer patients.

## CONCLUSIONS

We performed a comprehensive pCRCSC miRNA expression profile analysis and identified a novel pCRCSC-based miRNA signature that was intricately associated with the survival of patients with CRC. Further, the patients were categorized into high- and low-risk groups with substantially different clinical outcomes. Furthermore, the prognostic value of the pCRCSC-related miRNA signature was independent of other clinicopathological factors. Our study highlights the potential of pCRCSC-related miRNAs as alternative molecular markers, which could be used as promising therapeutic targets for CRC.

## MATERIALS AND METHODS

### Isolation, enrichment, and differentiation of CRCSC spheres from primary CRC

To isolate the primary CRCSC spheres, CRC samples were obtained from patients with primary colon adenocarcinoma who had undergone colon resection at the Department of Gastrointestinal Surgery, West China Hospital, Sichuan University. CRCSC spheres were isolated and expanded as described previously [3]. In brief, colon cancer tissues collected from surgical specimens were immediately minced on ice and suspended in the DMEM/F12 medium (HyClone, Logan, UT, USA). The tissue was mechanically and enzymatically dissociated, and the cell suspension was filtered. The dissociated single tumor cells were placed under stem cell conditions in serum-free DMEM/F12 medium supplemented with human recombinant EGF (PeproTech, Rocky Hill, NJ, USA) and bFGF (PeproTech) and cultured in ultra-low attachment plates (Corning, Corning, NY, USA). To obtain differentiated pCRCSCs, growth factors in the serum-free pCRCSC medium were removed and replaced with 20% FBS [3, 24].

### Xenograft experiments in a nude mouse model

Female nude mice (BALB/c strain, 4- to 6-week-old) were purchased from the Beijing Experimental Animal Centre of the Chinese Academy of Sciences (Beijing, China). The mice were housed under pathogen-free conditions, and the animal studies were performed according to the protocol approved by the Sichuan University Institutional Animal Care and Use Committee. The pCRCSC spheres and differentiated cells were trypsinized using 0.05% trypsin; 5 × 10^5^ cells were mixed with BD Matrigel (BD Biosciences, San Jose, CA, USA) at a 1:1 ratio and injected subcutaneously into the ventral wall of nude mice. Mice bearing the tumor were euthanized when the established criteria for the end-stage disease were reached, and the images were acquired.

### MiRNA isolation, miRNA microarray, and data analyses

MiRNAs of three pairs of CRCSCs and the corresponding differentiated cells were carefully isolated using “mirVana miRNA Isolation Kit” (Ambion, Darmstadt, Germany) following the manufacturer’s instructions. The purity and concentrations of miRNA samples were measured and determined spectrophotometrically with NanoDrop ND-2000c (Thermo Fisher Scientific, Inc., Wilmington, DE, USA).

Genome-wide miRNA profiles of three pairs of CRCSCs and their corresponding differentiated cells were analyzed using Agilent Human miRNA Microarray (V21) at Shanghai Biotechnology Corporation (Shanghai, China). The differential expression of miRNAs was identified using the Limma package in R 3.6.0 and selected based on adjusted *p*-values (*p* < 0.05). The absolute value of log2 fold change was >1.

### Prediction of target genes of differential miRNAs

MiRNA target genes were identified using miRWalk 2.0 (http://zmf.umm.uni-heidelberg.de/apps/zmf/mirwalk2/miRretsys-self.html), TargetScan 7.1 (http://www.targetscan.org), and miRDB (http://mirdb.org/miRDB/, v4.0). The target genes were further reduced by selecting those commonly predicted by all three databases.

### Gene ontology (GO) and kyoto encyclopedia of genes and genomes (KEGG) analyses

Biological process, cell component, molecular function, and KEGG pathways were annotated in the following way: the list of potential target transcripts for each pCRCSC-related miRNA was uploaded to the Database for Annotation, Visualization and Integrated Discovery (DAVID) (https://david.ncifcrf.gov) for functional annotation. A GO term and pathway were selected if miRNAs were significant (*p* < 0.05). The top 15 GO terms and pathways were visualized using the “ggplot2” package in R 3.6.0.

### pCRCSC-related miRNA-based prognostic model development

Level-3 data of miRNA-seq, mRNA-seq, including Illumina HiSeq and Illumina GA platforms, as well as the potential batch effects were removed using the "combat" function of "sva" package of R. The clinicopathological data of TCGA colorectal samples (COREAD) were obtained from UCSC Xena (https://xenabrowser.net/hub/). The adjuvant radiochemotherapeutic data were downloaded from GDC (https://portal.gdc.cancer.gov/projects). The TCGA colorectal samples were randomly divided into training and validation cohorts using the “caret” R package in R 3.6.0.

MiRNAs sharing both differential pCRCSC miRNAs and TCGA-COREAD miRNA datasets were considered as predictive prognostic markers for survival comparison, with the lowest log-rank *p* < 0.05. The candidate prognostic miRNA signature was identified using the multivariate Cox proportional hazard regression survival model. The prognostic risk scores for each patient were calculated using the formula based on the coefficient from the multivariate Cox proportional hazard regression model. The optimal cut-off was automatically selected by the X-tile software version 3.6.1 (Yale University School of Medicine, New Haven, CT, USA). The patients were subsequently divided into high- and low-risk groups using the optimal cut-off. Next, the area under the curve (AUC) was applied to assess the predictive accuracy of the pCRCSC miRNA prognostic model using the “survival ROC” package in R 3.6.0.

The differences in OS between the high and low pCRCSC-related miRNA scores were analyzed using the Kaplan–Meier (K–M) curve with a log-rank test based on the “survival” package in R 3.6.0.

### Statistical analyses

We compared different clinicopathological parameters in low- and high-risk groups using the *t*-test for continuous variables and χ^2^ test for categorical variables. The K–M method was used to compare the survival curves. The univariate and multivariate Cox regression model was used to study the prognostic significance of clinicopathological parameters and the CRCSC miRNA signature in the TCGA–COREAD data. All statistical tests were performed using the R software version 3.6.0. A *p*-value < 0.05 was considered significant.

### Ethics statement

This study was approved by the Independent Ethics Committee of the West China Hospital of Sichuan University and was performed in accordance with the Declaration of Helsinki (1983). Informed consent was obtained from all patients who provided samples.

## Supplementary Material

Supplementary Figures

Supplementary Table 1

Supplementary Table 2

Supplementary Table 3

Supplementary Table 4

Supplementary Table 5

Supplementary Table 6

Supplementary Table 7

Supplementary Table 8

Supplementary Tables 9 to 12
