# Supplementary material for: Expression profile, molecular functions, and prognostic significance of miRNAs in primary colorectal cancer stem cells
Source: Aging (Albany NY). 2021 Apr 1;13(8):12067–85. doi: 10.18632/aging.202914 (PMC8109135; doi:10.18632/aging.202914)
Supplement: Supplementary Figures [file aging-13-202914-s001.pdf]

SUPPLEMENTARY FIGURES

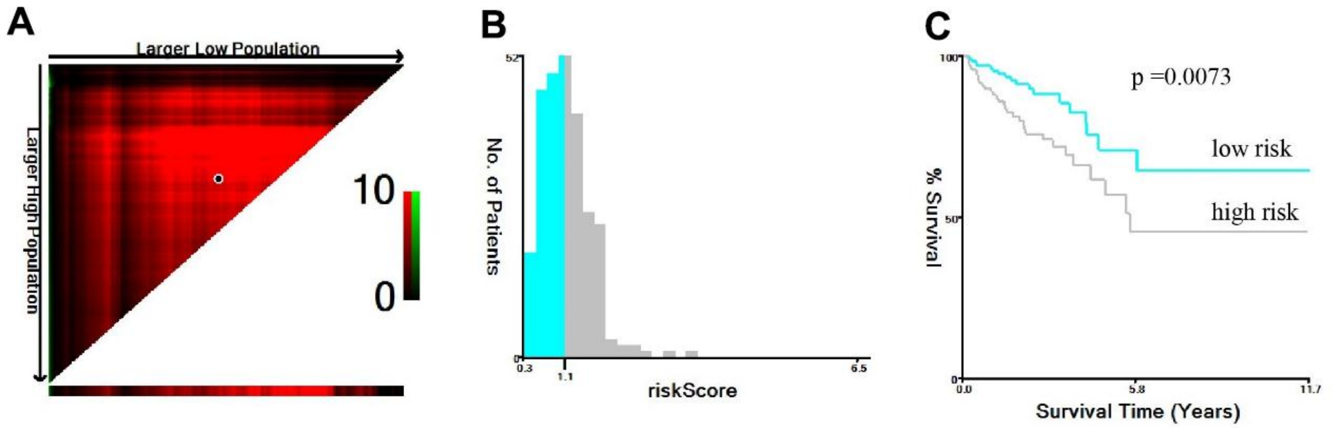

Supplementary Figure 1. X-tile plots of pCRCSCs-related miRNAs signature for optimal cut-off determination in training cohort. (**A**, **B**) The cut-off (IRS= 1.106) was optimized to separate low pCRCSCs-related miRNAs signature (blue) from high pCRCSCs-related miRNAs signature (gray) in the frequency histogram of training cohort. (**C**) Kaplan-Meier curve for testing the survival of sample subsets defined by optimized cutoff value of pCRCSCs-related miRNAs signature.

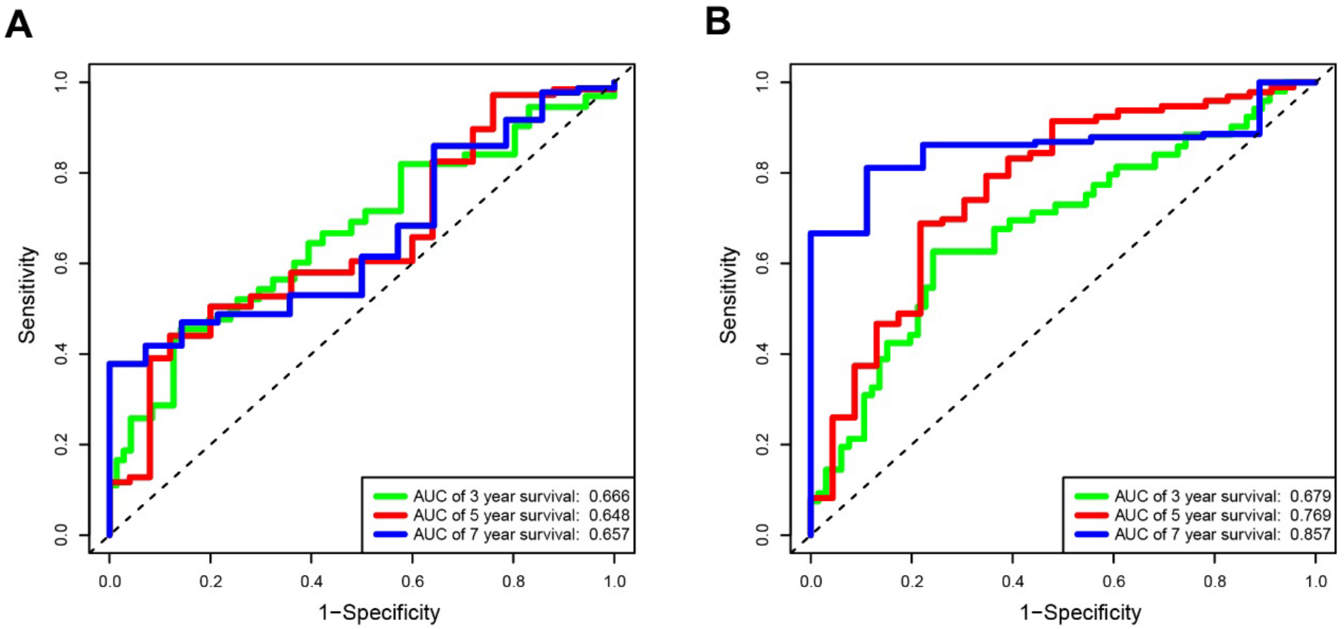

Supplementary Figure 2. Time-dependent ROC curves for OS-specific pCRCSC-related miRNA signature. (**A**) Time-dependent receiver operating characteristic curves at 3-, 5-, 7- years based on the pCRCSCs miRNAs signature in the training cohort. (**B**) Time-dependent receiver operating characteristic curves at 3-, 5-, 7- years based on the pCRCSCs miRNAs signature in the validation cohort.
