# Supplementary material for: Expression profile, molecular functions, and prognostic significance of miRNAs in primary colorectal cancer stem cells
Source: Aging (Albany NY). 2021 Apr 1;13(8):12067–85. doi: 10.18632/aging.202914 (PMC8109135; doi:10.18632/aging.202914)
Supplement: Supplementary Table 7 [file aging-13-202914-s008.pdf]

Supplementary Table 7. The KEGG pathway analysis of target genes of pCSCSC-related miRNAs.

Term	Count	%	p value	Genes
hsa00030:Pentose phosphate pathway	8	0.921659	8.55E-04	PGM2, GPI, RPE, PGM1, RPIA, PFKM, IDNK, PRPS1
hsa04520:Adherens junction	11	1.2672811	0.005685	PTPRJ, CDC42, SORBS1, BAIAP2, WASF2, MET, CTNND1, CDH1, WASL, INSR, TCF7L2
hsa04022:cGMP-PKG signaling pathway	18	2.0737327	0.006789	GNA13, KCNMA1, ATP1B3, PDE3A, ATP1A2, PPP1CB, VASP, KCNMB1, ATP2B1, EDNRB, ATP2B4, GNAQ, PLN, PDE5A, ADRA2A, INSR, MYLK, CALM1
hsa03040:Spliceosome	16	1.843318	0.006997	SRSF1, CHERP, SNRPB2, U2SURP, HNRNPU, SRSF3, HNRNPA3, SRSF2, SRSF5, TCERG1, SRSF7, SRSF6, SNRNP200, SNRPC, PRPF38B, RBM17
hsa04014:Ras signaling pathway	22	2.5345622	0.014486	FGFR2, PLD1, MET, KITLG, ARF6, KIT, FOXO4, STK4, CDC42, ETS2, RRAS2, GAB1, SOS2, PDGFRA, RALB, RAP1A, PDGFD, PRKACB, ABL1, INSR, GNG7, CALM1
hsa05200:Pathways in cancer	33	3.8018433	0.019088	GNA13, FGFR2, BID, E2F3, KITLG, FOXO1, EGLN1, CDH1, KIT, TCF7L2, PTEN, EDNRB, CDC42, BCL2, SOS2, RALB, TGFA, PRKACB, TPR, AXIN2, GNG7, PTGER4, EPAS1, MET, SKP2, ITGA2, FZD5, STK4, RAD51, HDAC2, GNAQ, PDGFRA, ABL1
hsa04152:AMPK signaling pathway	14	1.6129032	0.019246	PPP2R3A, PPP2R5A, FOXO1, ACACB, PFKM, PCK2, PPARGC1A, CPT1A, SLC2A4, PPP2CB, RAB14, RHEB, CAB39, INSR
hsa04972:Pancreatic secretion	11	1.2672811	0.03379	ATP2B1, KCNMA1, SLC26A3, CLCA1, ATP2B4, ATP1B3, SLC12A2, GNAQ, RAP1A, ATP1A2, SLC4A4
hsa04978:Mineral absorption	7	0.8064516	0.034055	SLC26A3, TRPM6, ATP1B3, SLC30A1, ATP1A2, SLC6A19, MT1G
hsa04931:Insulin resistance	12	1.3824885	0.037857	PPARA, SLC2A4, MLX, FOXO1, OGT, ACACB, PCK2, PTEN, PPARGC1A, INSR, PPP1CB, CPT1A
hsa04910:Insulin signaling pathway	14	1.6129032	0.043867	PRKAR2B, SORBS1, SLC2A4, SOS2, PHKA1, FOXO1, RHEB, PRKACB, ACACB, PCK2, PPARGC1A, INSR, PPP1CB, CALM1
hsa04261:Adrenergic signaling in cardiomyocytes	14	1.6129032	0.043867	PPP2R3A, ATP1B3, PPP2R5A, ATP1A2, PPP1CB, ATP2B1, ATP2B4, GNAQ, PLN, BCL2, PPP2CB, CAMK2D, PRKACB, CALM1
hsa04810:Regulation of actin cytoskeleton	19	2.1889401	0.045241	GNA13, FGFR2, ENAH, BAIAP2, PPP1R12B, WASF2, ITGA2, IQGAP2, PPP1CB, CDC42, DOCK1, EZR, CFL2, RRAS2, SOS2, PDGFRA, WASL, PDGFD, MYLK
hsa04922:Glucagon signaling pathway	11	1.2672811	0.048678	PPARA, GNAQ, PHKA1, CAMK2D, FOXO1, PRKACB, ACACB, PCK2, PPARGC1A, CPT1A, CALM1
hsa01212:Fatty acid metabolism	7	0.8064516	0.049232	ACOX1, ELOVL5, FADS1, HSD17B12, ACSL4, HADH, CPT1A
hsa01200:Carbon metabolism	12	1.3824885	0.049953	ME1, GPI, RPE, ME2, SUCLG2, ADPGK, RPIA, PFKM, IDNK, IDH3A, PC, PRPS1
