# Supplementary material for: Expression profile, molecular functions, and prognostic significance of miRNAs in primary colorectal cancer stem cells
Source: Aging (Albany NY). 2021 Apr 1;13(8):12067–85. doi: 10.18632/aging.202914 (PMC8109135; doi:10.18632/aging.202914)
Supplement: Supplementary Tables 9 to 12 [file aging-13-202914-s010.pdf]

Supplementary Table 9. Univariate screening of pCRCSC-related miRNAs for OS in the training cohort.

miRNAs	HR	HR.95L	HR.95H	p value
hsa-miR-142-3p	1.138066098	0.845905055	1.531134535	0.392881924
hsa-miR-182-5p	1.104825785	0.841234945	1.451009639	0.473486987
hsa-miR-183-5p	1.001026756	0.772377724	1.297363369	0.99381111
hsa-miR-196b-5p	0.879311673	0.729857866	1.059369303	0.175999667
hsa-miR-200c-5p	0.752123057	0.585874866	0.96554593	0.025412824
hsa-miR-223-3p	0.943087363	0.761021372	1.16871064	0.592357826
hsa-miR-338-3p	1.031115061	0.81146031	1.310228308	0.802055672
hsa-miR-338-5p	0.936520549	0.706315441	1.241755011	0.64864497
hsa-miR-345-5p	0.888078248	0.672958772	1.171963287	0.401630231
hsa-miR-3613-3p	1.135614061	0.730689762	1.764934125	0.571881692
hsa-miR-3614-5p	0.883264151	0.632935428	1.232598978	0.465361894
hsa-miR-3682-3p	1.18709029	0.769815217	1.830547545	0.437679937
hsa-miR-3940-3p	1.438622027	0.821532705	2.519234261	0.203279004
hsa-miR-4728-3p	1.061788613	0.716659991	1.573124038	0.764998501
hsa-miR-483-3p	0.896636456	0.764797104	1.0512029	0.17876179
hsa-miR-484	0.91859734	0.63191972	1.335329546	0.656421376
hsa-miR-5010-3p	1.100223917	0.656812919	1.842979384	0.716687824
hsa-miR-584-5p	0.83386517	0.679098374	1.023903382	0.082837655
hsa-miR-664b-3p	1.478618442	1.026965052	2.128906423	0.035462445
hsa-miR-664a-3p	1.106623781	0.731356495	1.674444955	0.631621212
hsa-miR-671-3p	0.96482922	0.658084789	1.41455241	0.854478312
hsa-miR-671-5p	1.086601465	0.761944892	1.549590733	0.646499192
hsa-miR-766-3p	0.931641917	0.643760078	1.348261087	0.707317911
hsa-miR-99b-5p	0.962351989	0.643240409	1.439774829	0.851897147
hsa-miR-494-3p	0.82117325	0.554024877	1.217139401	0.326458875
hsa-miR-598-3p	1.006536592	0.783515509	1.293038744	0.959340387

Supplementary Table 10. Multivariate screening of pCRCSC-related miRNAs for OS in the training cohort.

miRNA	coef	HR	HR.95L	HR.95H	p value
hsa-miR-200c-5p	-0.26960351	0.763682226	0.59521481	0.979832043	0.033990201
hsa-miR-664b-3p	0.383954209	1.468078215	1.003818613	2.147054873	0.047746837

Supplementary Table 11. Univariate analysis of pCRCSC-related miRNA signature for OS in the training cohort.

Parameters	HR	HR.95L	HR.95H	p value
Gender (Male vs Female)	1.409341027	0.767455353	2.588088183	0.268524673
Age (≤60 vs >60)	2.530791757	1.275012442	5.023407384	0.00794166
Location (RSCC vs LSCRC)	1.129825063	0.617866334	2.065988392	0.6918184
pTNM (II/III vs I)	5.209048044	0.707243848	38.36609056	0.105238693
pTNM (IV vs I)	20.28084515	2.677647553	153.6097159	0.003575243
MSI (MSS vs MSI)	1.015366899	0.451256094	2.284667076	0.970599452
RCT (WRCT vs RCT)	0.906118362	0.495940318	1.655542925	0.748521717
risk (High vs low)	2.17329531	1.160487595	4.070024121	0.015311349

Supplementary Table 12. Univariate analysis of pCRCSC-related miRNA signature for OS in the validation cohort.

Parameters	HR	HR.95L	HR.95H	p value
Gender (Male vs Female)	1.074137693	0.639349115	1.804603707	0.787025435
Age (≤60 vs >60)	2.510383032	1.396271857	4.51346415	0.002103413
Location (RSCC vs LSCRC)	1.754334544	1.049870147	2.931495576	0.031893283
pTNM (II/III vs I)	2.971275024	0.910809589	9.692997719	0.071057644
pTNM (IV vs I)	12.36401574	3.657776016	41.79285021	5.19E-05
MSI (MSS vs MSI)	1.252524391	0.534756327	2.933705079	0.604101677
RCT (WRCT vs RCT)	1.251448747	0.733429318	2.1353441	0.410642781
risk (High vs low)	3.709875215	2.114370252	6.509349107	4.87E-06
